# Perceived language legitimacy as a psychological judgment: a conceptual analysis of recognition, belonging, and self-worth

**DOI:** 10.3389/fpsyg.2026.1870995

**Published:** 2026-08-21

**Authors:** Shuzhao Wu, Jie Zeng, Xingpeng Zheng

**Affiliations:** 1 School of Foreign Languages, Fuzhou University of International Studies and Trade, Fuzhou, China; 2 School of Foreign Languages, Chengdu Normal University, Chengdu, China; 3 School of Digital Economy and Management, Chongqing Institute of Engineering, Chongqing, China

**Keywords:** conceptual analysis, linguistic evaluation, linguistic inequality, multilingualism, perceived language legitimacy, psychological judgment, self-worth, social recognition

## Abstract

This article develops perceived language legitimacy (PLL) as a theoretically refined framework for examining how languages and varieties come to be regarded as authoritative, valuable, or marginal within particular social contexts. Existing accounts of language attitudes, symbolic power, and language ideology have provided important explanations of linguistic evaluation and inequality. Less attention, however, has been given to the psychological appraisal through which speakers anticipate whether a language or variety will be accepted as a credible resource for knowledge, identity, authority, or public participation within a particular domain. PLL is conceptualized as a context-sensitive psychological judgment that complements these existing sociolinguistic and social-psychological accounts by explaining how historically established hierarchies, normative expectations, identity positions, and unequal distributions of symbolic value are interpreted by speakers. It provides a psychological link between socially organized linguistic hierarchies and speakers’ expectations of recognition or devaluation as language users. The article situates PLL in relation to established concepts including language attitudes, prestige, ideology, and identity. It further considers how legitimacy judgements may influence belonging, self-worth, educational uptake, public voice, and participation in multilingual societies. By focusing on this psychological interface, the article provides an account of how linguistic inequalities are experienced by speakers and translated into language-related expectations and actions.

## 1 Introduction

Languages are rarely evaluated solely on the basis of intelligibility. In multilingual settings, people do not only ask whether a language can be understood; they also judge whether it can credibly carry knowledge, authority, identity, or public consequence in a particular setting. A variety may be widely comprehensible and positively valued, yet still be treated as unsuitable for teaching, institutional communication, professional interaction, or public representation. Conversely, another may be accepted in these domains well beyond its immediate communicative utility. Language evaluation, therefore, cannot be explained by comprehension, usefulness, or personal preference alone. As demonstrated by extensive research in sociolinguistics, linguistic anthropology, and language ideology, such evaluations are shaped by social positioning, relations of power, and historically developed understandings of linguistic value. Such evaluations also involve judgments about which languages belong in socially consequential spaces, which speakers are entitled to use them there, and whose linguistic contributions will be taken seriously. These questions are sociolinguistic and institutional, but they are also psychological because they shape how speakers anticipate recognition, evaluate their own linguistic resources, and decide whether to participate, accommodate, remain silent, or withdraw.

The question of linguistic legitimacy is not new. Bourdieu’s (1991) account of linguistic markets and symbolic power demonstrated how institutions and social relations establish particular linguistic forms as legitimate and endow their speakers with unequal authority. Heller (1996) subsequently examined how legitimate languages and legitimate speaker positions are produced within multilingual education, while Reagan (2016) clarified the social, cultural, and political dimensions of language legitimacy. More recent work has shown that legitimacy is not determined by official policy alone but may also be locally constructed through interactional norms and practiced language policies (Bonacina-Pugh, 2020). Linguistic anthropological and critical sociolinguistic research has further shown that legitimacy is negotiated through processes of indexicality, voice, authorisation, and speakers’ lived experience of language use (Blommaert, 2010; Busch, 2017; Bucholtz and Hall, 2005). Beyond sociolinguistics, institutional legitimacy has been theorized as the perceived congruence of an entity or practice with socially established norms and values (Suchman, 1995), whereas psychological research has examined why authorities and social arrangements are accepted as having a justified right to govern conduct (Tyler, 2006). Recognition theory has further explained how being recognized or misrecognized affects self-confidence, self-respect, and social esteem (Honneth, 1995). The present article therefore does not claim to discover linguistic legitimacy as a previously unidentified phenomenon. Rather, it seeks to provide a psychologically oriented account of how legitimacy-related evaluations are formed, experienced, and translated into expectations of recognition and participation across different social domains.

Existing approaches illuminate different aspects of linguistic inequality. Language attitudes address evaluative orientations, while theories of symbolic power, language ideology, recognition, and institutional legitimacy explain how linguistic value is socially produced, negotiated, and experienced across social contexts. These traditions have examined how linguistic legitimacy is produced, negotiated, and challenged across historical, institutional, and interactional settings. Building on these perspectives, the present framework focuses on a complementary psychological question: how individuals form situated judgments about whether a particular linguistic resource is likely to receive recognition or rejection within a specific domain.

Critical perspectives further show that linguistic evaluation is shaped by racialised expectations, unequal speaker positions, and struggles over participation (Stroud, 2001; Flores and Rosa, 2015). These approaches have provided important accounts of how linguistic legitimacy, appropriateness, and speaker recognition are negotiated within unequal social conditions. Building on these insights, PLL focuses on the psychological process through which individuals interpret such conditions and form expectations about whether their own linguistic practices, or those of others, will be recognized as appropriate, credible, and socially consequential. This helps explain why official recognition may coexist with everyday avoidance, or why strong language identification may coexist with doubts about a language’s capacity to support authoritative action.

This article develops perceived language legitimacy (PLL) as a theoretically refined, middle-range framework for addressing this explanatory gap. PLL is defined as a context-sensitive psychological judgment through which individuals perceive whether a language, variety, accent, repertoire, or communicative practice is socially authorized to perform a particular function within a specified domain, and whether its users are likely to receive recognition rather than correction, dismissal, or devaluation. This definition highlights four defining characteristics that clarify the nature of PLL as a psychological judgment. First, the judgment has a defined linguistic object rather than referring to social legitimacy in general. This feature specifies what the judgment is about, rather than constituting a dimension of PLL itself. Second, it is domain-specific: the same language may be strongly authorized in family life but weakly authorized in in another. Among families that actively maintain Sámi, for example, the language may be used mostly or exclusively with children, whereas access to Sámi-medium education remains limited, particularly in Sweden, where provision is confined to a small number of schools and extends only to Year 6 (Lloyd-Smith et al., 2023). This contrast illustrates why PLL may vary across social settings. Third, it is relational because legitimacy depends on who uses the language, before which audience, and for what purpose. This relational feature highlights that legitimacy judgments concern not only linguistic forms but also their users and audiences. Fourth, it is anticipatory: speakers form expectations about how their linguistic conduct will be received, and these expectations may subsequently influence emotion, motivation, self-regulation, and action. PLL is therefore not an inherent property of a language, a synonym for official status, or a stronger form of positive attitude. It identifies the situated judgment through which wider linguistic hierarchies are experienced by individuals as either enabling or constraining recognized participation.

Perceived language legitimacy is intended to complement, rather than replace, established accounts of language attitudes, symbolic power, ideology, recognition, or identity. Rather, it seeks to integrate insights from these traditions by specifying how socially organized evaluations of language may be experienced at the psychological level as expectations of linguistic authorization, credibility, and possible action.

A further question concerns how legitimacy-related judgments are formed and enacted psychologically. Speakers encounter institutional policies, educational practices, public discourse, interpersonal feedback, and representations of linguistic groups as cues concerning what forms of language use will be recognized. These cues are cognitively appraised in relation to the speaker, audience, purpose, and anticipated consequences of language use, building on sociolinguistic insights that language meanings are socially mediated rather than individually generated. The framework proposes that legitimacy judgments may be affectively experienced as security, pride, anxiety, shame, or hesitation. This proposed relationship is informed by research on language-related affect and lived linguistic experience, although it has not yet been tested directly in relation to PLL (Busch, 2017; Park, 2021; Ponsonnet, 2022). Through repeated exposure and social feedback, external linguistic hierarchies may also become internalized through processes of social learning and self-evaluation as expectations that guide self-monitoring, code choice, participation, and withdrawal. Recent research on perceived language policy similarly suggests that the psychological and behavioral effects of institutional language arrangements depend partly on how those arrangements are interpreted by language users (Wang, 2026). PLL is thus neither a private preference detached from context nor a direct reflection of formal policy. It is a socially formed appraisal through which structural conditions become affectively lived, motivationally relevant, and behaviorally consequential.

**FIGURE 1:**
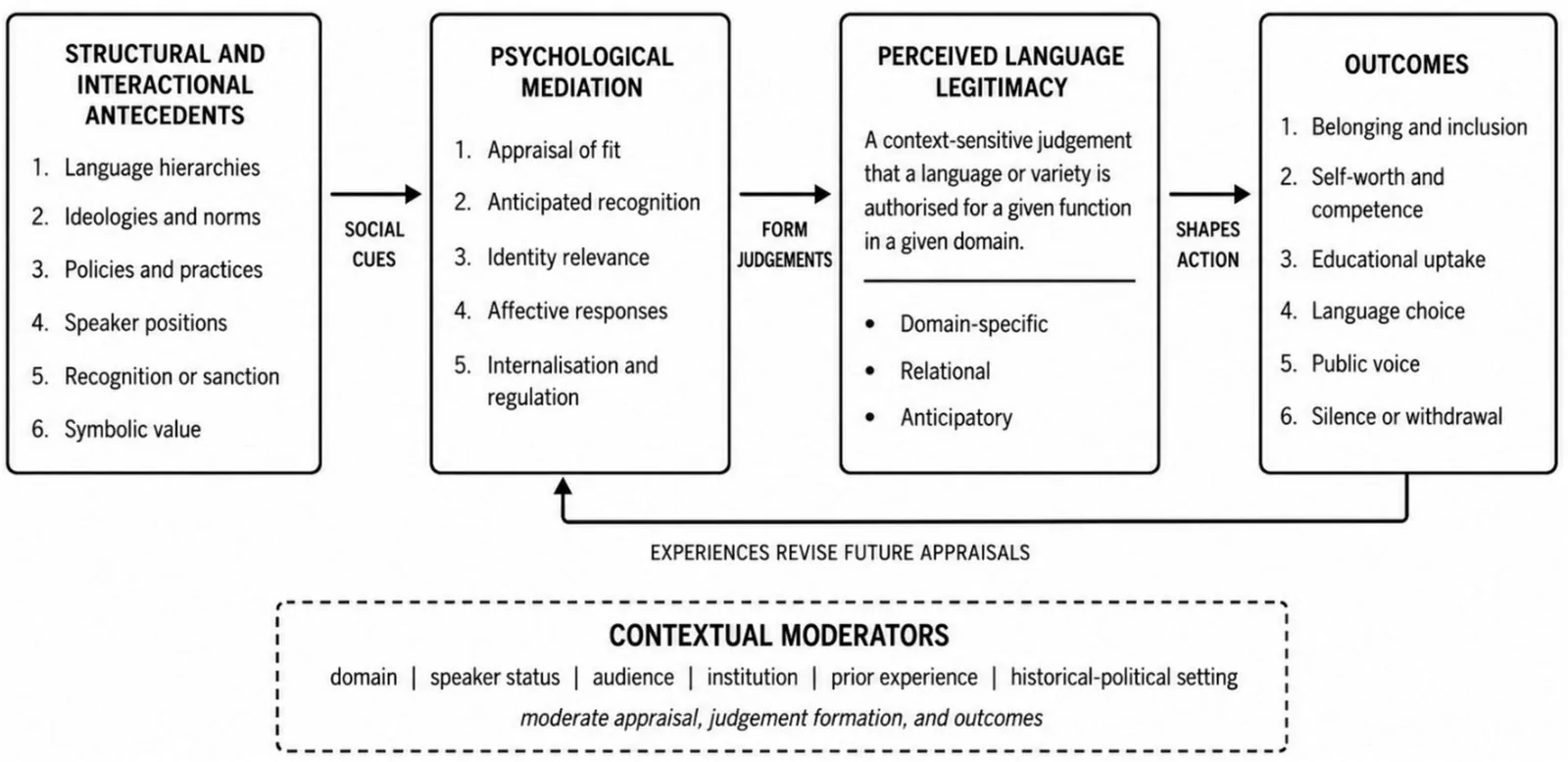
An explanatory framework of perceived language legitimacy (PLL) as a context-sensitive psychological judgment.

The article pursues four related aims. First, it reconstructs the conceptual genealogy of linguistic legitimacy and explains how a psychological account of its perceived form can complement existing sociolinguistic and linguistic-anthropological approaches. Second, it provides a more rigorous specification of PLL by identifying its defining characteristics, clarifying its domain-specific and relational nature, and establishing its analytical relationship with language attitudes, prestige, ideology, identity, and formal recognition. Third, it develops a theoretical account of the cognitive, affective, motivational, and internalization processes through which legitimacy judgements connect symbolic language hierarchies with belonging, self-worth, educational uptake, public voice, and language-related behavior. Fourth, it proposes a preliminary multidimensional model and an empirical research agenda through which PLL may subsequently be examined using qualitative, survey-based, experimental, longitudinal, and comparative designs. Figure 1 presents the resulting explanatory framework, tracing pathways from structural and interactional antecedents through appraisal and internalization processes to PLL judgements and their psychological and behavioral outcomes, while recognizing that these pathways are moderated by domain, speaker position, audience, and prior experience. The broader purpose is to clarify the psychological process through which linguistic legitimacy becomes understood, experienced, and enacted in social life.

## 2 Conceptual genealogy and the theoretical need for PLL

The conceptual foundations of PLL extend beyond language-attitude research. Bourdieu’s (1991) account of legitimate language, linguistic markets, and symbolic power established that linguistic resources acquire value through historically structured relations of authority rather than through linguistic form alone. Within such markets, particular languages and varieties become associated with education, expertise, institutional credibility, and social advancement, while others are confined to less valued domains. Subsequent sociolinguistic work has shown that legitimacy is not determined solely by formal status. It may also be produced, negotiated, and contested within multilingual institutions and everyday interaction (Heller, 1996; Reagan, 2016; Bonacina-Pugh, 2020). These studies make clear that languages, speakers, and communicative practices are not equally authorized across social settings. More recent sociolinguistic and linguistic-anthropological research has further demonstrated that processes of authorization and differentiation are negotiated through everyday interaction and through speakers’ attempts to position themselves within unequal linguistic orders (Gal and Irvine, 1995; Bucholtz and Hall, 2005; Blommaert, 2010; Busch, 2017).

Related theories illuminate further dimensions of this problem. Language-attitude research explains how speakers evaluate languages, varieties, and their users in relation to status, solidarity, competence, attractiveness, and trustworthiness (Garrett, 2010). Language ideology identifies the socially shared assumptions through which linguistic differences are linked to authority, morality, correctness, authenticity, and social belonging (Silverstein, 1979; Woolard and Schieffelin, 1994). Importantly, language ideology research has also examined how such assumptions are reproduced, contested, and embodied in everyday practices, shaping speakers’ experiences of appropriateness, belonging, and exclusion.

Recent phenomenological and affect-oriented approaches in linguistic anthropology further demonstrate that language ideologies are not only systems of social meaning but also lived experiences through which speakers understand their own linguistic possibilities, identities, and social positions. Busch (2017), for example, conceptualizes linguistic experience as a lived relationship between speakers and their repertoires, highlighting how feelings of attachment, insecurity, and displacement emerge from encounters with socially valued and devalued ways of speaking. Ochs (1996) further shows that linguistic resources participate in the socialization of personhood by connecting forms of expression with culturally recognizable identities, roles, and expectations. More recent affect-oriented research has also emphasized that emotions are not merely consequences of linguistic inequality but constitute an important dimension through which language-ideological processes are experienced and negotiated (Park, 2021, 2026; Ponsonnet, 2022). Together, these studies show that linguistic hierarchies are not only reproduced through institutions and discourse but are also encountered in speakers’ everyday experiences. Building on this insight, PLL focuses on the specific psychological judgment through which speakers evaluate whether a linguistic resource is expected to receive recognition, authorization, and acceptance for a particular function within a particular domain.

Recognition theory explains why recognition and misrecognition matter for self-confidence, self-respect, and social esteem (Honneth, 1995, 2020). Institutional and psychological legitimacy research, meanwhile, examines why authorities, organizations, and social arrangements are accepted as consistent with shared norms or as entitled to regulate conduct (Suchman, 1995; Tyler, 2006). Raciolinguistic perspectives further demonstrate that linguistic evaluations may be shaped by racialised expectations concerning speakers and listeners (Flores and Rosa, 2015), whereas linguistic citizenship foregrounds speakers’ capacity to challenge dominant language regimes and create alternative spaces of participation (Stroud, 2001).

Language-attitude research captures favorable and unfavorable evaluations of linguistic resources, but such evaluations do not necessarily establish whether a language or variety is perceived as authorized for a particular social function. Symbolic-power and language-ideology approaches explain how unequal linguistic value is produced and naturalized, while recognition, raciolinguistic, and citizenship perspectives show how such valuations affect speakers’ positions and possibilities for participation (Flores and Rosa, 2015; Stroud, 2001). The present article builds on these insights by examining the psychological appraisal through which socially organized evaluations become expectations of credibility, recognition, or constraint within particular domains.

Perceived language legitimacy is developed to specify this intermediate psychological level. It does not seek to replace sociolinguistic accounts of legitimacy, but to identify the psychological appraisal process through which socially produced linguistic values become personally meaningful and behaviorally relevant. It refers to a context-sensitive judgment that a language, variety, accent, repertoire, or communicative practice is socially authorized to perform a specified function within a particular domain, and that its users are likely to be recognized as credible participants when using it for that purpose. PLL therefore identifies the psychological interface through which these wider processes of language evaluation, symbolic power, and social recognition become relevant to anticipated reception and possible action.

This distinction helps explain cases in which symbolic recognition and practical authorization diverge. For example, Sámi languages in Northern Europe have received institutional support through revitalization policies, yet research indicates that formal recognition alone does not guarantee equal authority across education, particularly in domains requiring advanced knowledge production and academic participation, or in everyday social life (Lloyd-Smith et al., 2023). Similarly, localized English varieties, such as Singapore English, may function as important markers of national or local identity while Standard English remains privileged in some educational and professional settings (Jenkins, 2015; Rose and Galloway, 2019). Such cases cannot be fully explained by formal status, prestige, positive attitudes, or identity attachment considered separately. They require attention to the situated psychological judgment through which linguistic resources become experienced as credible, usable, and socially consequential.

Perceived language legitimacy should thus be understood as a theoretically refined, middle-range framework rather than as a wholly unprecedented concept. Its distinctive contribution lies in specifying the point at which socially organized linguistic hierarchies are perceived and interpreted as permissions or constraints on recognized participation. In this sense, PLL represents not a rejection of earlier work, but an analytical refinement that connects structural inequality, social recognition, psychological appraisal, and language-related action.

## 3 A psychological process account of PLL

Perceived language legitimacy is best understood as the outcome of a socially situated psychological process rather than as a private opinion or a direct reflection of formal language status. This view builds on developments in language-attitude research that have moved beyond simple evaluative preferences toward context-sensitive interpretations of linguistic meaning, social positioning, and speaker evaluation. Speakers encounter languages within environments in which different linguistic resources have already acquired associations with education, authority, intimacy, expertise, ethnicity, locality, and social mobility. These socially available meanings do not determine judgment mechanically. Rather, consistent with sociolinguistic and social-psychological accounts of language evaluation, they provide interpretive resources through which speakers evaluate the possible consequences of language use. PLL emerges when such resources are interpreted in relation to a particular linguistic practice, domain, audience, purpose, and anticipated outcome of interaction.

The first mechanism is cognitive appraisal. Appraisal theories propose that situations acquire psychological significance through evaluations of relevance, congruence with goals and norms, likely consequences, and possibilities for control (Scherer and Moors, 2019). Applied to language, speakers may assess whether a linguistic resource fits the demands of a setting, whether its use is compatible with audience and institutional expectations, and whether it is likely to support or undermine credibility. Such appraisals may involve questions such as whether the language will be recognized in that domain, whether its users will be taken seriously, and whether its use may invite correction, dismissal, ridicule, or sanction. PLL is the judgment that emerges from these appraisals: a context-specific expectation that a linguistic resource is, or is not, socially authorized to perform the intended function in that setting.

A second mechanism concerns social identity and self-categorization. Social Identity Theory and Self-Categorization Theory suggest that people interpret themselves partly through contextually salient group memberships (Tajfel and Turner, 1979; Turner et al., 1987). Because languages, accents, and repertoires often index nationality, ethnicity, region, class, education, or professional membership, legitimacy judgments may concern not only whether a language belongs in a setting, but also whether people positioned like the speaker are recognized as entitled to use it there. This perspective is consistent with sociocultural linguistic research showing that linguistic practices contribute to identity positioning and the negotiation of social meaning (Bucholtz and Hall, 2005). PLL therefore concerns how identity-related meanings become relevant to expectations of recognition or exclusion in particular contexts.

The same linguistic resource may therefore be judged differently depending on who uses it, before which audience, and for what purpose. Empirical research on accent discrimination offers indirect support for the psychological consequences proposed here. Among native and non-native speakers of French, perceived accent discrimination was negatively associated with language confidence (Freynet and Clément, 2019). Gluszek and Dovidio (2010) similarly found that speaking with a non-native accent was associated with a weaker sense of social belonging, partly through perceived communication difficulties. These studies do not test PLL directly, but they suggest that repeated linguistic devaluation may be related to speakers’ confidence and sense of belonging. Possible effects on identity continuity and distinctiveness remain theoretical propositions derived from Identity Process Theory (Breakwell, 1986) and require direct empirical examination.

These appraisals and identity-based interpretations may, in turn, have affective and motivational consequences. Empirical research on accent discrimination provides relevant, although indirect, support for this proposed pathway. Perceived accent discrimination has been associated with lower language confidence among both native and non-native speakers of French (Freynet and Clément, 2019), while non-native-accented speakers in the United States have reported communication difficulties and a weaker sense of social belonging (Gluszek and Dovidio, 2010). Research in workplace settings has also shown that non-native English speakers may experience heightened stereotype threat in interactions with native speakers, with consequences for their perceptions of conflict and its outcomes (Kim et al., 2022). Building on these findings, the PLL framework proposes that anticipated recognition may foster security, ease, and confidence, whereas anticipated devaluation may be associated with anxiety, hesitation, or linguistic insecurity. These responses are treated here as possible consequences of PLL rather than as components of the construct itself, and their relationship with PLL remains to be tested directly. Self-Determination Theory further provides a basis for expecting legitimacy judgments to influence motivation through perceived competence, autonomy, and relatedness (Ryan and Deci, 2000, 2020).

Through repeated social feedback and processes of socialization, these processes may become internalized. Drawing on a university student’s retrospective linguistic autobiography, Busch (2017) describes how she felt linguistically deficient after moving from a village school to a secondary school in a regional capital, where her rural vernacular was negatively contrasted with the High German spoken by classmates from higher social strata. Her subsequent efforts to adopt High German led her to monitor her own speech closely and to experience it as inauthentic. Such experiences may contribute to durable expectations about which ways of speaking are acceptable and how closely one’s speech must be monitored. The framework further proposes that recognition and successful participation may revise earlier expectations and strengthen PLL. Legitimacy judgments may, in turn, shape self-regulation through code choice, rehearsal, accent monitoring, participation, silence, or avoidance. These proposed behavioral pathways require direct empirical examination.

**FIGURE 2:**
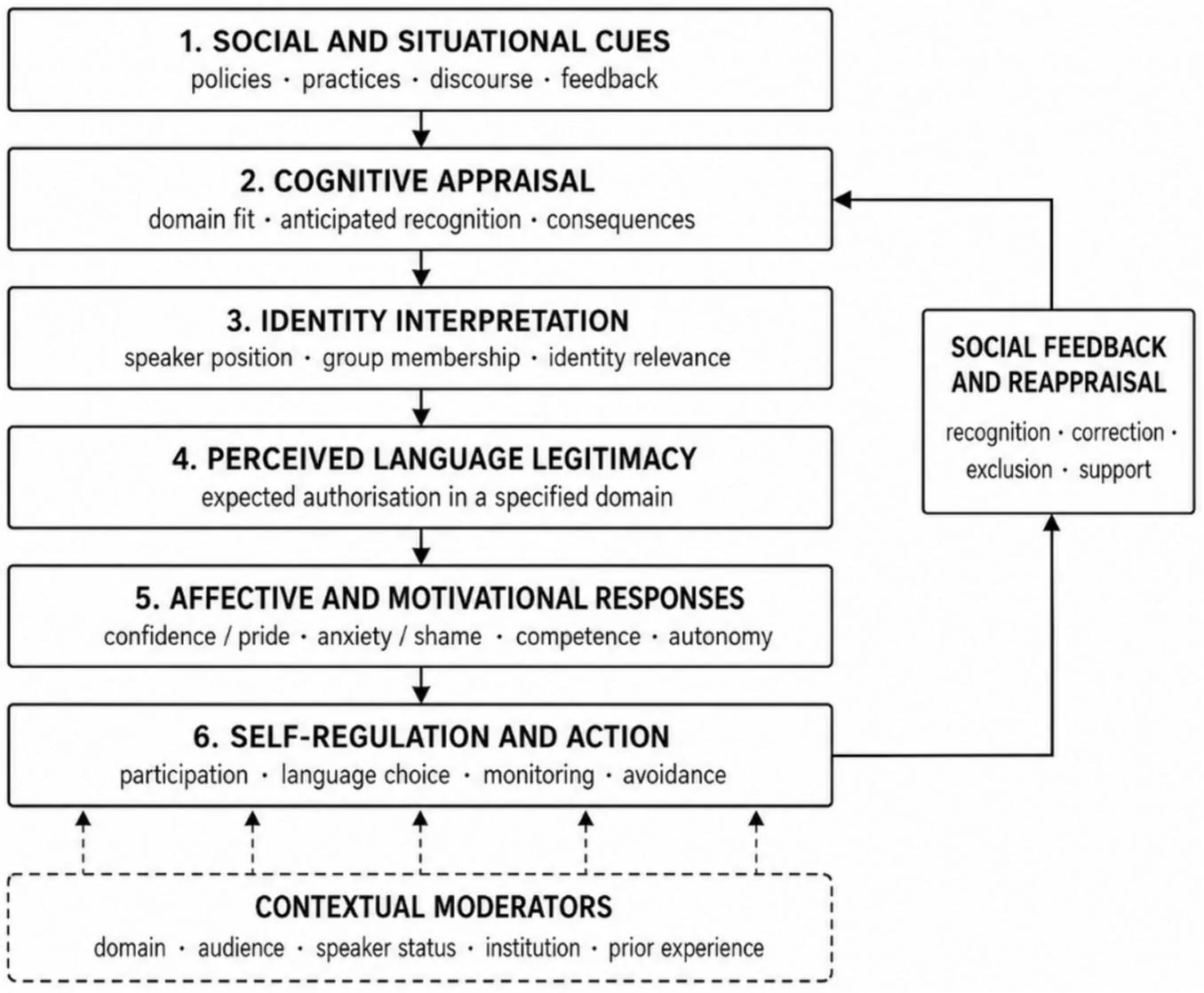
Psychological processes underlying perceived language legitimacy (PLL).

The process is therefore recursive rather than linear. This recursive account is also consistent with contemporary research on norm dynamics, which shows that social norms are maintained and transformed through interaction among collective expectations, identity processes, behavior, and social feedback (Gelfand et al., 2024). Rather than treating social feedback as a purely external force, the PLL framework specifies how such feedback is interpreted by individuals and incorporated into subsequent judgments of linguistic authorization and participation. Social and institutional cues shape appraisal, while subsequent recognition or sanction may reinforce or revise later judgments. The influence of these processes is likely to vary according to domain, audience, speaker position, institutional context, and prior experience. Figure 2 represents this psychological process by showing how social cues are interpreted through appraisal and identity processes, the proposed pathways through which PLL may be associated with affective and motivational responses, and how behavior and social feedback may contribute to the revision of later judgments.

## 4 Conceptual boundaries and analytical position of PLL

The usefulness of PLL depends on specifying not only how it differs from neighboring constructs, but also how its conceptual structure relates to the social conditions from which it emerges and the outcomes that may follow from it. PLL is not intended as an umbrella term for all forms of linguistic evaluation, inequality, identity, or recognition. Nor is it proposed as a replacement for established accounts of language attitudes, prestige, ideology, symbolic power, recognition, or legitimacy. It identifies a more delimited psychological judgment: how speakers perceive whether a particular linguistic resource, when used by particular speakers for a particular purpose, is expected to be socially authorized within a specified domain.

At the conceptual level, PLL has four defining characteristics. First, it concerns a specific linguistic object, such as a language, variety, accent, repertoire, or communicative practice. Second, it is domain-sensitive rather than a fixed property of the linguistic resource itself: the same resource may be authorized in one setting but questioned in another. Third, it is relational because legitimacy judgments involve not only linguistic forms but also speakers, audiences, purposes, and social positions. Fourth, it is anticipatory because it concerns expectations about whether future language use will receive recognition, acceptance, correction, dismissal, or sanction. These characteristics describe the nature of the judgment itself rather than constituting separate dimensions of PLL.

Perceived language legitimacy also varies according to contextual conditions. These conditions include the social domain in which language use occurs, the institutional setting, the speaker’s social position, and the audience to whom communication is directed. Such conditions do not form components of PLL; rather, they shape how legitimacy judgments are formed in particular situations. Consistent with sociolinguistic research showing that legitimacy is negotiated beyond official policy and institutional recognition, official recognition may influence PLL, but legal status and perceived authorization are not equivalent.

### 4.1 Core dimensions of PLL

Within this conceptual framework, the four defining characteristics describe the conceptual nature of PLL, whereas the construct may be further specified as a multidimensional judgment comprising three analytically distinguishable but interrelated dimensions. These dimensions represent possible ways of operationalizing PLL rather than additional properties of the construct itself. They are theoretically derived from existing discussions of linguistic authorization, social recognition, speaker positioning, and anticipated social evaluation rather than generated through inductive procedures.

The first is functional-domain authorization: whether a specified linguistic resource is perceived as capable of performing a particular function within a given domain, such as teaching, assessment, professional communication, administration, identity expression, or public debate. This dimension builds on sociocultural linguistic research showing that speaker positions and linguistic practices are mutually constructed through interaction, while focusing specifically on how such positioning is psychologically interpreted as entitlement or constraint. The second is speaker entitlement: whether a particular speaker or category of speakers is perceived as socially entitled and sufficiently credible to use that linguistic resource for the intended function. The third is anticipated social reception: whether such language use is expected to receive recognition and serious engagement rather than correction, dismissal, ridicule, exclusion, or sanction.

The three dimensions are related but should not be conflated. A language may be authorized for a function in principle while particular speakers are not regarded as equally entitled to use it. For example, a standard language may be strongly authorized in professional communication, while speakers with a marked regional or non-native accent may nevertheless be treated as less credible or encounter accent-based devaluation (Freynet and Clément, 2019; Gluszek and Dovidio, 2010; Kim et al., 2022). Conversely, a speaker may be accepted as a legitimate participant while a particular variety within that speaker’s repertoire remains excluded from formal use. Formal recognition may also support functional authorization without producing an expectation of positive social reception in everyday practice.

Accordingly, PLL concerns the relational configuration of a linguistic resource, its intended function, the position of its user, and its anticipated reception. General judgments of attractiveness, usefulness, prestige, identity value, or legal recognition do not constitute PLL unless they also involve expected authorization within a specified context.

The three dimensions provide a preliminary conceptual structure rather than a validated psychometric model. Future empirical research will need to determine whether they are empirically separable, whether they form a higher-order PLL construct, and whether their relative importance varies across languages, speakers, institutions, and social domains. At the conceptual level, however, they specify the principal content of a legitimacy judgment while preserving the distinction between PLL, its social antecedents, and its psychological and behavioral consequences.

### 4.2 PLL and language attitudes

Language attitudes concern evaluative orientations toward languages, varieties, accents, and their users. Contemporary language-attitude research has moved beyond simple positive–negative evaluations and examines how such orientations are shaped by social meanings and contexts. They commonly include judgments of status, solidarity, competence, attractiveness, and trustworthiness (Garrett, 2010). PLL intersects with this tradition but addresses a more specific question: not simply whether a linguistic resource is evaluated positively or negatively, but whether it is expected to be accepted as capable of performing a particular function within a particular domain.

The distinction means that evaluative approval does not necessarily translate into perceived authorization. Singlish, or Singapore Colloquial English, provides a concrete example. In interviews with Singaporean undergraduates living overseas, Lee and Ahn (2021) found that Singlish served as a marker of identification and solidarity, yet participants also associated its use with impropriety and embarrassment in some contexts. The variety could therefore carry positive identity value without being perceived as equally appropriate across different social settings. Language attitudes may therefore contribute to PLL, but evaluative valence alone cannot establish whether speakers expect a linguistic resource to support credible and consequential participation.

### 4.3 PLL, prestige, and symbolic power

Prestige refers primarily to the comparative social ranking of linguistic resources. It explains why some languages and varieties are associated with education, refinement, authority, or mobility, whereas others are linked to locality, intimacy, or lower status. Symbolic power provides a broader structural account of how these rankings are produced and naturalized through institutions, linguistic markets, and authorized forms of speech (Bourdieu, 1991).

Perceived language legitimacy differs from prestige and symbolic power in that it focuses on the psychological appraisal of linguistic value within a particular situation. Prestige concerns the relative status attached to linguistic resources, whereas symbolic power concerns the social processes through which such status relations are produced and maintained. PLL examines how speakers interpret these relations when judging whether a linguistic resource is likely to be accepted, recognized, or challenged in a specific domain.

Prestige and symbolic power may therefore shape the conditions under which PLL develops, but they do not determine legitimacy judgments in every context. For example, in an induction classroom for newly arrived immigrant children in France, languages other than French were treated as legitimate under the locally established “practiced language policy,” even though they were not authorized by top-down language policies (Bonacina-Pugh, 2020). Symbolic power and prestige are therefore important social antecedents of PLL, but PLL identifies the situated judgment through which speakers translate socially organized linguistic value into expectations of recognition, acceptance, or sanction.

### 4.4 PLL and language ideology

Language ideology refers to socially shared beliefs and assumptions through which linguistic differences are connected to wider ideas of correctness, civility, authenticity, nationhood, morality, and authority (Silverstein, 1979; Woolard and Schieffelin, 1994). Ideologies provide the interpretative frameworks through which particular languages and speakers become intelligible as educated, local, modern, backward, authentic, or out of place, while also being reproduced, challenged, and transformed through everyday interaction and social practice.

Within this broader sociolinguistic framework, PLL focuses on the psychological appraisal through which individuals interpret the implications of such ideological meanings for a particular act of language use. Language ideologies may shape expectations about which languages are appropriate for education, professional communication, or public expression. PLL concerns whether individuals perceive a particular linguistic resource as likely to receive recognition, acceptance, or devaluation within a specific domain. In this sense, language ideology provides a socially organized interpretative context for legitimacy judgments, while PLL specifies the situated psychological process through which such meanings become relevant to anticipated participation and social reception.

### 4.5 PLL and general legitimacy

General legitimacy theory examines whether authorities, organizations, institutions, or social arrangements are perceived as normatively justified or entitled to regulate conduct (Suchman, 1995; Tyler, 2006). This tradition provides an important foundation for PLL by demonstrating that legitimacy is not simply conferred through formal status or institutional power, but depends on how social arrangements are interpreted and accepted by those involved.

Perceived language legitimacy extends this insight into the domain of language by shifting attention from the legitimacy of social arrangements to the perceived authorization of linguistic resources and practices. Whereas general legitimacy research addresses the perceived appropriateness and acceptability of authorities, institutions, or social arrangements, PLL focuses on how individuals judge whether a linguistic resource or practice is likely to be recognized as appropriate, credible, and authorized within a particular domain. The two constructs therefore operate at related but distinct analytical levels: general legitimacy concerns the perceived rightfulness of social arrangements, whereas PLL concerns the psychological judgment through which language users anticipate whether their linguistic practices will be accepted in a specific context. PLL consequently applies the broader insight of perceived legitimacy to language-related participation without treating linguistic legitimacy as an inherent property of a language itself.

### 4.6 PLL and recognition

Recognition theory concerns the interpersonal and institutional recognition of persons and social groups and explains why recognition matters for self-confidence, self-respect, and social esteem (Honneth, 1995). Sociolinguistic research has further shown that recognition is often mediated through language practices, as speakers’ voices, identities, and forms of participation may be valued or marginalized through unequal processes of evaluation. PLL is connected to recognition because linguistic practices provide one important route through which speakers seek social acceptance, credibility, and participation. Nevertheless, the two concepts should not be conflated.

Recognition concerns broader social and moral relations through which persons and groups are acknowledged or denied social value. PLL focuses more specifically on the psychological judgment that a linguistic resource is expected to support or constrain recognized participation within a particular domain. A speaker may seek recognition as a competent student, professional, citizen, or group member, while PLL concerns whether the language through which that claim is made is expected to support or undermine recognition. In this sense, recognition helps explain why linguistic authorization matters for the self, whereas PLL specifies how individuals anticipate the possibility of recognition through particular forms of language use.

### 4.7 PLL and identity

Identity concerns how speakers understand themselves, how they are positioned by others, and how language contributes to group membership, belonging, continuity, and distinction. Sociocultural linguistic research has demonstrated that identities are not simply possessed by speakers but are continuously negotiated through linguistic practices, interaction, and social positioning (Bucholtz and Hall, 2005). PLL and identity frequently interact, but they address different analytical questions. Identity concerns the meanings and positions that speakers construct through language, whereas PLL concerns whether the linguistic resources associated with those meanings are expected to receive recognition in a particular domain.

A strong linguistic identity does not necessarily result in strong PLL. The Singlish example discussed above illustrates this point: the variety can serve as a marker of Singaporean identity and solidarity, yet its use remains contested in formal educational and professional settings, where Standard English is often preferred (Jenkins, 2015; Rose and Galloway, 2019; Lee and Ahn, 2021). Identity is therefore neither a component nor a synonym of PLL. Instead, identity provides one interpretative resource through which legitimacy judgments may be formed, while PLL concerns the anticipated authorization of the linguistic practices through which identities are expressed and negotiated. It may shape legitimacy judgments, and PLL may in turn influence whether a language-based identity can be expressed securely and publicly sustained.

### 4.8 The analytical position of PLL

These distinctions locate PLL within a broader conceptual and psychological sequence. Language ideologies, institutional practices, symbolic power, prestige hierarchies, and prior experiences provide social and interpretative conditions. Speakers appraise these conditions in relation to a linguistic resource, domain, audience, and purpose. PLL is the resulting situated psychological appraisal of expected linguistic authorization, formed through individuals’ interpretation of these conditions within specific domains. This judgment may then influence affective experience, motivation, self-regulation, language choice, participation, and withdrawal. These relationships are proposed by the present framework and remain to be tested directly, although related research on accent discrimination provides indirect evidence linking linguistic devaluation with reduced language confidence, communication difficulties, and weaker social belonging (Freynet and Clément, 2019; Gluszek and Dovidio, 2010).

Perceived language legitimacy should therefore not be treated as a composite label for its antecedents and consequences. Ideology, prestige, symbolic power, institutional practice, and prior experience help shape legitimacy judgements, whereas belonging, self-worth, emotion, participation, and withdrawal may follow from them. PLL occupies an intermediate analytical position between socially organized conditions and potential psychological or behavioral outcomes: it captures the judgment through which linguistic value becomes personally relevant in a specific context. Table 1 summarizes these distinctions by identifying the primary focus, analytical level, and relationship of each neighboring construct to PLL.

**TABLE 1:** Conceptual and analytical boundaries between perceived language legitimacy (PLL) and related constructs.

Construct	Primary focus	Main analytical level	Relationship to PLL
Language attitudes	Favorable or unfavorable evaluations of languages, varieties, and speakers	Evaluative and dispositional	Attitudes may inform PLL, but positive evaluation does not establish domain-specific authorization.
Language prestige	Comparative status and symbolic ranking of linguistic resources	Social and relational	Prestige may increase the likelihood of strong PLL, but rank does not determine authorization in every domain.
Symbolic power	Institutional production and reproduction of legitimate linguistic value	Structural and institutional	Symbolic power provides conditions from which PLL develops; PLL concerns how those conditions are interpreted in a particular situation.
Language ideology	Shared assumptions linking language to authority, correctness, identity, morality, and social order	Discursive, historical, and cultural	Ideology supplies socially organized meanings and interpretative resources; PLL concerns how individuals appraise the expected recognition of particular linguistic practices within those meanings.
General legitimacy	Perceived rightfulness of authorities, institutions, and social arrangements	Institutional and psychological	General legitimacy provides the broader logic of justified authority; PLL applies that logic to a linguistic resource and its domain-specific function.
Recognition	Social acknowledgment and valuation of persons and groups	Interpersonal, institutional, and moral	Recognition explains why PLL matters to the self; PLL specifies whether language is expected to support recognized participation.
Language identity	Self-positioning, group membership, belonging, and continuity through language	Individual, relational, and collective	Identity may shape and be affected by PLL, but identification does not establish whether a language is authorized in a given domain.
Perceived language legitimacy	Functional-domain authorization, speaker entitlement, and anticipated social reception	Situated psychological judgment	PLL links socially organized linguistic value to expectations that a specified linguistic resource and its users will be recognized as credible within a particular domain.

Collectively, these distinctions position PLL as a domain-specific psychological judgment of expected linguistic authorization rather than as a synonym for evaluation, status, identity, or recognition. The framework therefore distinguishes between the social conditions that shape legitimacy judgments, the psychological appraisal through which they are formed, and the possible consequences that may follow from them. With these conceptual boundaries established, the next section considers the consequences of PLL for belonging, self-worth, educational uptake, and public voice.

## 5 Potential implications of PLL for belonging, self-worth, educational uptake, and public voice

The PLL framework proposes that legitimacy judgments may have implications for belonging, self-worth, educational uptake, and public voice. These relationships have not yet been tested directly as consequences of PLL. The discussion below therefore draws on related empirical and theoretical research to identify plausible pathways for future investigation.

First, PLL may have implications for a sense of belonging. Belonging depends not only on identification, but also on whether one’s linguistic resources are recognized as fitting the space one inhabits. Speakers may feel a strong attachment to a language and still experience limited belonging when that language is treated as inappropriate or out of place in valued settings. The framework therefore proposes that, when a language is recognized as appropriate and admissible, speakers may experience their own linguistic presence as ordinary rather than provisional. Empirical research on non-native accents provides relevant evidence for this proposed relationship. Across two studies in the United States, Gluszek and Dovidio (2010) found that speakers with non-native accents reported a weaker sense of social belonging, and that this association was mediated by perceived communication difficulties. PLL helps explain why belonging in multilingual settings is not simply a matter of access to multiple languages, but also depends on whether speakers perceive those languages as socially authorized to count. In psychological terms, legitimacy supports a sense that one’s way of speaking can be present without apology, correction, or defensive explanation. This observation is consistent with phenomenological accounts of linguistic experience, which show that speakers’ sense of belonging is shaped by whether their linguistic repertoires are experienced as recognized or devalued within particular social contexts (Busch, 2017). Related research on non-native accents has linked perceived communication difficulties and linguistic devaluation with a weaker sense of social belonging (Gluszek and Dovidio, 2010).

Second, PLL may be relevant to self-worth. Languages are tied not only to communication, but also to competence, credibility, and social value. When a speaker’s repertoire is repeatedly positioned as secondary, inappropriate, or deficient, the issue is rarely linguistic alone. It may also affect the speaker’s sense of adequacy and entitlement to be heard. In this way, legitimacy marks the point at which symbolic hierarchy becomes lived self-evaluation. Building on research on linguistic insecurity and affective experiences of language inequality, PLL specifies the evaluative process through which perceived linguistic devaluation may become connected to feelings of inadequacy, hesitation, or reduced confidence (Busch, 2017; Park, 2021; Freynet and Clément, 2019; Gluszek and Dovidio, 2010). Its effects may appear not only in overt shame, but also in hesitation, self-correction, accommodation, or the feeling that one’s preferred language is somehow insufficient for serious purposes. PLL therefore provides a way of examining how broader linguistic inequalities may become relevant to speakers’ evaluations of their own competence, legitimacy, and social position.

Third, PLL may help explain aspects of educational uptake. A language may be officially supported and pedagogically useful, yet still encounter resistance if learners, teachers, or institutions do not regard it as a proper medium of knowledge, authority, or advancement. Conversely, a dominant language may retain educational authority not simply because of utility, but because it is more widely perceived as the rightful language of serious learning. This is especially evident where local or non-dominant varieties are granted symbolic recognition but denied full pedagogical authority. Such tensions have been documented in multilingual educational settings where formal recognition has not always been matched by equal pedagogical authority or provision (Heller, 1996; Cann, 2004; Lloyd-Smith et al., 2023). In such cases, the problem is less explicit rejection than limited legitimacy. The variety may be acknowledged, yet not fully authorized for textbooks, examinations, or formal instruction. PLL draws attention to the psychological dimension of this gap: whether learners and educators perceive the language as capable of carrying legitimate knowledge and academic value. Educational uptake is therefore not only a matter of policy design or instructional effectiveness; it also depends on whether a language is psychologically accepted as capable of conveying knowledge, supporting assessment, and fostering academic aspiration.

Finally, PLL may have implications for public voice. To speak in public is not only to communicate, but also to claim recognition, relevance, and the right to participate. When a language is treated as fully legitimate, speakers can use it to intervene, represent, and be heard as socially consequential actors. When it is treated as marginal or only locally valid, public speech may become narrowed or risky even where the language remains meaningful in private life. Consistent with sociolinguistic accounts of voice and linguistic inequality, legitimacy helps explain why some languages continue to dominate not only because they are useful, but because they are widely experienced as the voices that properly count. Public voice thus reveals the behavioral consequences of legitimacy judgments: speakers may participate, remain silent, shift codes, or limit their claims depending on whether their language is perceived as authorized in the public sphere. This proposed relationship between PLL and public voice remains to be examined directly through empirical research on language choice, participation, and self-silencing in public settings.

Taken together, these consequences show how PLL provides a psychological link between symbolic language hierarchies and social action. The framework therefore proposes that PLL may shape whether speakers feel that their linguistic resources can support belonging, sustain self-worth, enter education, and carry public consequence. These proposed relationships provide directions for future empirical research.

## 6 Implications for multilingual societies

The concept of PLL offers a psychological perspective for understanding recognition in multilingual societies. Recognition involves more than the formal naming, protection, or celebration of languages; it also concerns whether speakers perceive those languages as capable of carrying knowledge, identity, authority, and public consequence. From a theoretical psychological perspective, this means that recognition must be understood not only as an institutional arrangement, but also as a lived judgment through which speakers assess whether their linguistic resources can legitimately support self-expression, learning, participation, and social action. As synthesized in Table 2, the legitimacy of a language is often highly domain-specific, revealing a stark contrast between private acceptance and public authorization. Recent work on multilingual development similarly emphasizes that language dominance, attitudes, identities, and investment are shaped within different multilingual ecologies and by both explicit and implicit language policies (Siemund, 2023).

**TABLE 2:** Domain-specific variation in perceived language legitimacy (PLL) across social domains.

Social domain	Dominant language/standard variety	Local or marginalized variety
Family and private life	May be functionally accepted, though sometimes felt as formal or affectively distant.	May be associated with intimacy, family memory, and heritage, as illustrated by minoritized languages such as te reo Māori in Aotearoa New Zealand or Sámi languages in Nordic communities (Barrett-Walker et al., 2020; Lloyd-Smith et al., 2023)..
Education	Commonly institutionally authorized as a principal medium of knowledge, assessment, and academic authority, such as English in many international higher education contexts.	May receive symbolic recognition but limited pedagogical authority, as illustrated by Welsh-medium education in Wales or Sámi language education in Nordic countries (Cann, 2004; Lloyd-Smith et al., 2023).
Institutional life	Commonly authorized for administration, law, expertise, and formal recognition, such as national standard languages used in government and professional settings.	May remain secondary or restricted in institutional practice, particularly where formal recognition has not been matched by routine use in administrative or professional domains.
Public voice	May be treated as the unmarked or authoritative medium of official political, academic, and media communication.	May be locally meaningful while receiving less authority in formal public communication, as illustrated by continuing debates over the place of Singlish in formal settings (Jenkins, 2015; Rose and Galloway, 2019; Lee and Ahn, 2021).

The contrasts presented here are illustrative, domain-sensitive tendencies rather than universal properties of dominant or minoritized languages. Their expression may vary according to the linguistic resource, speaker, audience, institution, and social context.

Official recognition does not necessarily guarantee perceived legitimacy across all domains. Lloyd-Smith et al. (2023), for example, compared Sámi language policies and language practices in Sweden and Norway using survey data from 5,416 Sámi and non-Sámi participants across 20 northern municipalities. They found that Sámi language use had declined substantially across three generations, that only a small proportion of Sámi participants were highly fluent and used the language with their children, and that Sámi use was most common in the home. Language use and proficiency were higher in Norway, where the policy environment was more favorable, but remained limited in both countries. These findings do not measure PLL directly, but they demonstrate that formal recognition and policy support can coexist with restricted opportunities for learning and sustained use. The PLL framework interprets this gap as a possible context in which speakers may come to doubt whether their linguistic resources will receive full recognition across valued domains. Related research on accent discrimination further suggests that experiences of linguistic devaluation may be associated with reduced language confidence and weaker social belonging (Freynet and Clément, 2019; Gluszek and Dovidio, 2010).

This is especially important in multilingual education. The success of educational reform cannot be fully understood through curriculum design or policy support alone. It also depends on whether a language is perceived as a proper medium of knowledge, discipline, and advancement. For instance, Welsh has achieved substantial institutional recognition and a well-established presence in Welsh-medium education, while research has highlighted the continuing importance of expanding Welsh-medium provision in higher education and professional domains (Cann, 2004). A language may be formally included in schooling yet remain weakly perceived as legitimate if teachers, learners, or institutions continue to treat it as unsuitable for serious academic work. Conversely, dominant languages often retain their authority not only because of utility, but because they are more deeply naturalized as perceived rightful languages of education (May, 2014; García and Li, 2014). Similar tensions can be seen in the case of Singlish, whose local social significance coexists with continuing debate over its place in formal educational and professional communication (Jenkins, 2015; Rose and Galloway, 2019). For theoretical psychology, the important point is that educational acceptance involves more than policy compliance or instrumental calculation; it also involves psychological judgments about whether a language can legitimately carry knowledge, aspiration, credibility, and future opportunity.

The concept also has implications for language policy and linguistic inequality. Contemporary language-policy research increasingly treats policy as a process involving formulation, implementation, interpretation, monitoring, and evaluation rather than as a formal declaration alone (Gazzola et al., 2024). Such perspectives have shown that the effects of language policy are mediated through institutional practices, local interpretations, and everyday language experiences rather than determined by official statements alone. PLL complements this line of research by specifying the psychological dimension of such mediation: how speakers interpret policy environments and social practices as signals of possible linguistic authorization or constraint.

The revitalization of te reo Māori further illustrates the importance of sustained social conditions beyond formal recognition. Although Māori has gained official recognition and substantial educational support in Aotearoa New Zealand, research suggests that its continued development depends on opportunities for learning, intergenerational transmission, and sustained everyday use rather than institutional support alone (Barrett-Walker et al., 2020). This highlights the distinction between formal recognition and lived legitimacy: a language may be institutionally supported while still requiring sustained social conditions that enable meaningful use across different contexts. Research on family language policy similarly demonstrates how childhood language experiences, social norms, opportunities, and language-related anxiety can shape later language retention intentions (Pagé and Noels, 2024). Together, these findings suggest that policy outcomes depend not only on institutional arrangements but also on how linguistic possibilities are perceived, evaluated, and internalized by language users.

The framework therefore offers a useful vocabulary for future work on multilingual education, localized English, language policy, and linguistic justice. It shifts attention from whether a language is merely liked, tolerated, or officially recognized to whether it is perceived as entitled to matter in socially consequential domains. By connecting symbolic hierarchies with speakers’ situated judgments and language-related behavior, PLL helps explain how linguistic inequality becomes meaningful in everyday experience.

## 7 Toward empirical operationalization

Perceived language legitimacy remains a conceptual framework rather than a validated psychometric construct. Future research should therefore examine whether the proposed dimensions of functional-domain authorization, speaker entitlement, and anticipated social reception can be empirically identified, refined, or revised across different linguistic contexts. Any future measures should remain perception-based and domain-specific, while clearly specifying the linguistic resource, speaker, audience, purpose, and institutional setting under consideration.

Functional-domain authorization may be assessed through context-sensitive judgments about whether a language, variety, accent, or communicative practice is considered credible for activities such as teaching, assessment, professional communication, administration, knowledge production, or public debate. Speaker entitlement may be examined through perceptions of whether particular speakers are regarded as competent, credible, and socially entitled to use that resource in a given setting. Anticipated social reception may be captured through expectations of recognition, trust, serious engagement, correction, ridicule, dismissal, exclusion, or sanction.

Future scale development could draw on established approaches in language attitude research, sociolinguistic perception studies, and social psychological measurement. It may combine theoretically derived items with interviews, focus groups, cognitive interviewing, and scenario-based tasks across different multilingual settings. Survey studies could then use exploratory and confirmatory factor analysis to examine dimensionality, reliability, measurement invariance, and convergent and discriminant validity. In particular, PLL should be distinguished empirically from language attitudes, prestige, language identity, linguistic insecurity, and general institutional trust.

Vignette-based experiments could manipulate language variety, speaker identity, domain, audience, or institutional endorsement to examine how legitimacy judgments are formed rather than assuming that legitimacy is inherent in linguistic forms. Longitudinal and experience-sampling approaches could further examine how PLL changes through policy reform, educational intervention, migration, or repeated experiences of recognition and correction. Comparative and mixed-methods designs could further test whether PLL operates similarly across linguistic, educational, professional, and national contexts, while also linking self-reported judgments to observed language choice, participation, self-monitoring, or withdrawal.

## 8 Conclusion

In this article, we have argued that language evaluation cannot be adequately understood through intelligibility, utility, or preference alone. Drawing together insights from sociolinguistics, linguistic anthropology, and psychology, this article has developed PLL as a middle-range framework for examining whether a linguistic resource is expected to perform a specified function credibly within a particular domain and whether its users are likely to be recognized as legitimate participants.

Perceived language legitimacy is neither purely individual nor simply institutional. It emerges through socially organized hierarchies, institutional practices, interactional experience, appraisal, and internalization. Its contribution does not lie in identifying an entirely new phenomenon, but in specifying a psychological level at which broader processes of linguistic evaluation, authorization, and recognition become experienced as expectations of credibility and possible action. Rather than replacing existing theories of language attitudes, symbolic power, language ideology, recognition, or identity, PLL complements them by clarifying how socially organized meanings are interpreted by individuals within particular domains. It distinguishes whether a language is present, valued, or officially recognized from whether speakers expect it to sustain consequential participation in a particular setting. This distinction offers a focused basis for future empirical research on how linguistic inequalities are perceived, internalized, and negotiated in multilingual life.
